# Recent Advances in Biosensor Technology for Potential Applications – An Overview

**DOI:** 10.3389/fbioe.2016.00011

**Published:** 2016-02-16

**Authors:** S. Vigneshvar, C. C. Sudhakumari, Balasubramanian Senthilkumaran, Hridayesh Prakash

**Affiliations:** ^1^VIT University Chennai Campus, Chennai, India; ^2^Department of Animal Biology, University of Hyderabad, Hyderabad, India; ^3^School of Life Sciences, University of Hyderabad, Hyderabad, India

**Keywords:** biosensors, electrochemical, nanomaterials, fluorescence-tag, bioelectronics, polymer, microbes, diseases

## Abstract

Imperative utilization of biosensors has acquired paramount importance in the field of drug discovery, biomedicine, food safety standards, defense, security, and environmental monitoring. This has led to the invention of precise and powerful analytical tools using biological sensing element as biosensor. Glucometers utilizing the strategy of electrochemical detection of oxygen or hydrogen peroxide using immobilized glucose oxidase electrode seeded the discovery of biosensors. Recent advances in biological techniques and instrumentation involving fluorescence tag to nanomaterials have increased the sensitive limit of biosensors. Use of aptamers or nucleotides, affibodies, peptide arrays, and molecule imprinted polymers provide tools to develop innovative biosensors over classical methods. Integrated approaches provided a better perspective for developing specific and sensitive biosensors with high regenerative potentials. Various biosensors ranging from nanomaterials, polymers to microbes have wider potential applications. It is quite important to integrate multifaceted approaches to design biosensors that have the potential for diverse usage. In light of this, this review provides an overview of different types of biosensors being used ranging from electrochemical, fluorescence tagged, nanomaterials, silica or quartz, and microbes for various biomedical and environmental applications with future outlook of biosensor technology.

## Introduction

The term “biosensor” refers to powerful and innovative analytical device involving biological sensing element with wide range of applications, such as drug discovery, diagnosis, biomedicine, food safety and processing, environmental monitoring, defense, and security. The first biosensor invented by Clark and Lyons (1962) to measure glucose in biological samples utilized the strategy of electrochemical detection of oxygen or hydrogen peroxide (Fracchiolla et al., 2013; Turner, 2013) using immobilized glucose oxidase electrode. Since then, incredible progress has been made (Turner, 2013) both in technology and applications of biosensors with innovative approaches involving electrochemistry, nanotechnology to bioelectronics. Considering the phenomenal advances in the field of biosensors, this review is aimed to introduce various technical strategies, adopted for developing biosensors in order to provide fundamental knowledge and present scientific scenario of biosensor technology. With the emphasis on the research tools that demonstrate how the performance of biosensors evolved from the classical electrochemical to optical/visual, polymers, silica, glass, and nanomaterials to improve the detection limit, sensitivity, and selectivity. Interestingly, microbes and bioluminescence (Du et al., 2007) also contributed largely for label-based biosensors, while label-free biosensors involved usage of transistor or capacitor-based devices and nanomaterials. Biosensors provide a basis to understand technological improvement in the instrumentation involving sophisticated high-throughput machines for quantitative biologists and portable qualitative or semi-quantitative devices for non-specialists. Finally, current research trends, future challenges, and limitations in the field are highlighted. The present review is divided to various subsections describing two major technical strategies followed by various types of biosensor devices ranging from electrochemical, optical/visual, polymers, silica, glass, and nanomaterials. These devices were developed for specific purposes and an overview of these will provide readers a comprehensive data on biosensor devices and their applications.

## Technical Strategies

The technical strategies (Turner, 2013) used in biosensors are based on label-based and label-free detection. Label-based detection is mainly dependent upon the specific properties of label compounds to target detection. These types of biosensors though reliable but often require combination of specific sensing element fabricated with immobilized target protein. On the other hand, label-free method (Citartan et al., 2013; Sang et al., 2015) allows detecting the target molecules that are not labeled or difficult to tag. Recent interdisciplinary approaches of biotechnology with bioengineering, electrical and electronics engineering paved way for developing label-free biosensors for various detection methods with wide range of applications in the fields of medicine and environmental science.

## Electrochemical Biosensors

Classical discovery of glucometer using glucose oxidase-based biosensors (Clark and Lyons, 1962) is first in the line of discovery of electrochemical biosensors. Glucose biosensors are widely popular among hospitals or diagnostic clinics as these are essential for diabetic patients for periodic monitoring of blood glucose. However, glucose biosensors are often having drawbacks due to instable enzyme activity or inhomogeneity (Harris et al., 2013) for which further calibration is essential. In fact, these potential drawbacks lead to the invention of range of biomolecules (Turner, 2013; Wang et al., 2013) having differential electrochemical properties, which paved way to discover more viable glucose biosensors. In recent times, electrochemical biosensors (Wang et al., 2014) are typically prepared by modifying the surface of metal and carbon electrodes using biomaterials, such as enzyme, antibody, or DNA. Biosensor’s output signal is usually generated upon specific binding or catalytic reactions of biomaterials (Wang et al., 2014) on the surface of electrode. The need for the discovery of electrochemical sensors became indispensible for clinical diagnosis of diseases (Gruhl et al., 2013) where in early detection or monitoring seems essential. In this context, development of non-enzymatic biosensors is often considered by using synthetic materials in place of proteins. Interestingly, various types of biomolecules have differential electrode stability and selectivity, which ultimately contribute to the development of new types of electrochemical biosensors for various purposes. Several types of electrochemical biosensors were developed based on their usage. As explained above, glucose biosensors (Harris et al., 2013) had undergone rapid evolution from the time of development. In this perspective, Wang et al. (2014) reviewed the progress of ferroceneboronic acid (FcBA) and ferrocene (Fc)-modified boronic acids for biosensor development due to the presence of binding site (i.e., a boronic acid moiety) and an electrochemically active part (i.e., an Fc residue). FcBA and its derivatives possess unique property of binding to 1,2- or 1,3-diol residues of sugars leading to the formation of cyclic boronate ester bonds. The redox properties of FcBA-sugar adduct differ from free FcBA forming a basis for electrochemical detection. In addition to this, boronic acids have affinity for binding to Fe^−^ ions, which gives an additional advantage for the development of non-conventional ion-selective electrodes using F^−^ ion. Hydrocarbon chains present in the polypeptide chain of HbA1c can be measured using FcBA based electrochemical detection. Major limitation in using this method is the requirement of immobilization of FcBA derivatives (Wang et al., 2014) on the surface of electrodes as these derivatives are added in sample solutions as reagents. Use of polymers and/or silver electrodes with suitable modification of FcBA derivatives may lead to improvement of FcBA electrochemical sensor for biomedical fields including diabetes diagnosis wherein glucose measurement will be supplemental.

Electrochemical biosensor to assess the levels of antioxidants and reactive oxygen species in physiological systems (Mello et al., 2013) is another modern invention. Major application in this line is the detection of uric acid as primary end product of body fluid purine metabolism (Erden and Kilic, 2013), which provide diagnostic tool for various clinical abnormalities or diseases. However, it is essential to develop a cost-effective and sensitive method. Electrochemical-based approach for measurement of uric acid oxidation likewise for glucose quantification seems ideal. However, resemblance of uric acid in terms of oxidation with ascorbic acid poses major experimental hurdle to develop highly sensitive electrochemical biosensor. To overcome this, scientists have developed amperometric detection-based biosensor (Erden and Kilic, 2013), which possesses the ability to measure both reduction and oxidation potentials. Considering the cost and reproducibility for this procedure, it is important to immobilize or screen print the enzyme on electrodes or else on nanomaterial-based electrodes, which are ideal for development of disposable, selective, cost-effective and sensitive uric acid biosensors for routine analysis. In this regard, recent advances in 3D bioprinting (Turner, 2013) with an aim to create biosensors with live cells encapsulated in 3D microenvironments. In the same line, a new wireless mouth-guard biosensor to detect salivary uric acid level in real-time and continuous fashion was developed (Kim et al., 2015), and the technology can be extended to wearable monitoring tools for diverse health and fitness applications. Electrochemical biosensors have been successfully used for hormone measurements (Bahadir and Sezginturk, 2015) yet, its perspective needs to be reviewed in detail. Another potential area of technology development in biosensors relies on targeting nucleic acids. It is well known that cellular miRNA expression is an ideal biomarker for the diagnosis of onset of disease and targeting those improves efficacy of gene therapy for genetic disorders. Usually, miRNAs are detected by northern blotting, microarray, and polymerase chain reaction. Modern technology provides ideal electrochemical biosensors for miRNA detection based on label-free detection involving guanine oxidation subsequent to the hybrid formation involving the miRNA (Hamidi-Asl et al., 2013) and its inosine substitute capture probe. All these inventions are due to modern approaches of biofabrification for promoting electrochemical based biosensor technology in biomedicine.

Environmental monitoring (Long et al., 2013; Verma and Bhardwaj, 2015) is another important aspect wherein biosensor technology is required for rapid identification of pesticidal residues to prevent health hazards. Traditional methods, such as high-performance liquid chromatography, capillary electrophoresis and mass spectrometry, are effective for the analysis of pesticides in the environment (Verma and Bhardwaj, 2015) yet, there are limitations for instance complexity, time-consuming procedures, requirement of high-end instruments, and operational capabilities. Hence, simple biosensors seem to have tremendous advantages yet, it is cumbersome to develop unified one for analyzing various classes of pesticides. To this end, certain enzyme-based biosensors (Pundir and Chauhan, 2012; Verma and Bhardwaj, 2015) were developed to understand the physiological impact of pesticides in the environment, food safety, and quality control. For this purpose, acetylcholinesterase (AChE) inhibition-based biosensors were developed (Pundir and Chauhan, 2012). Over the last decade or two, for rapid analysis this technique got improved further with recent developments in AChE inhibition-based biosensors including immobilization methods and other different strategies for fabrication. Similarly, piezoelectric biosensors have been developed (Marrazza, 2014) for detecting the organophosphate and carbamate pesticidal impact in the environment. Organochlorine pesticides are known to affect ecosystem (Senthilkumaran, 2015) where in the pesticides like endosulfan cause considerable damage. Indeed, such pesticides alter the reproductive system of male and female fishes differentially (Senthilkumaran, 2015) and considering these facts, invention of biosensors to test the aquatic ecosystem will have greater significance in view of biomagnification. To cope up with the demand, electrochemical biosensor underwent revolution (Turner, 2013; Verma and Bhardwaj, 2015) with rapid advances in fabrication and use of nanomaterials or quartz or silica. It is important to place special emphasis for selection of receptors for biosensor development, the use of different transduction techniques and fast screening strategies for applications of biosensor in food, and environmental safety and monitoring. To enable this, biosensor fabrication seems to be important and the advancements in this field were categorically explained below.

## Optical/Visual Biosensors

As explained above, environmental or biomedical applications demand the development of simple, swift, and ultra-sensitive biosensors. This could be possible with immobilizers (Guo, 2013; Ogi, 2013; Turner, 2013; Peng et al., 2014; Shen et al., 2014) ranging from gold, carbon-based materials, silica, quartz, or glass. In fact, the incorporation of gold nanoparticles or quantum dots with the use of micro-fabrication provides new technology (Schneider and Clark, 2013) for the development of highly sensitive and portable cytochrome P450 enzyme biosensors for certain purpose. Furthermore, fiber-optic chemical sensors have lot of relevance in various fields, such as drug discovery, biosensing, and biomedicine. More recently, hydrogels, used as DNA-based sensor, are emerging materials for immobilization usage with fiber-optic chemistry (Dias et al., 2014). Compared to other materials, immobilization in hydrogels occurs in 3D which allows high loading capacity of sensing molecules. Hydrogels (polyacrylamide) are hydrophilic cross-linked polymers (Khimji et al., 2013) and can be made into different forms for immobilization ranging from thin films to nanoparticles. Hydrogels are considered as a simple substrate for DNA immobilization with other advantages, such as entrapment, controlled release, analyte enhancement, and DNA protection. These features are unique to hydrogels compared to other materials which offer biomolecular immobilization (Khimji et al., 2013). Furthermore, good optical transparency of hydrogels provides convenient strategy for visual detection. Detailed methods for immobilizing DNA biosensors (Khimji et al., 2013) in monolithic polyacrylamide gels and gel microparticles are often considered as technical advancement in the field of biosensor technology. Single molecule detection has also been developed using electrochemical oxidation of hydrazine for DNA detection (Kwon and Bard, 2012).

## Silica, Quartz/Crystal and Glass Biosensors

Recent methods in the development of biosensors resulted in the use of silica, quartz or crystal and glass materials due to their unique properties. Among these, silicon nanomaterials have greater potential for technological advances in biosensor applications due to their biocompatibility, abundance, electronic, optical, and mechanical properties. Furthermore, silicon nanomaterials have no toxicity which is an important prerequisite of biomedical and biological applications. Silicon nanomaterials (Peng et al., 2014; Shen et al., 2014) offer wide range of applications ranging from bioimaging, biosensing and cancer therapy. Furthermore, fluorescent silicon nanomaterials have long-term applications in bioimaging. Interestingly, silicon nanowires in combinations with gold nanoparticles provide hybrids which are used (Shen et al., 2014) as novel silicon-based nano-reagents for effective cancer therapies. Covalent attachment of thiol-modified DNA oligomers on silica or glass provides DNA films for better usage in UV spectroscopy and hybridization methods (Khimji et al., 2013). Despite lot of advantages to use silicon nanoparticles, widespread challenges such as development of large-scale low-cost production methods and biocompatibility after biomolecular contact needs to be evaluated. Resolving these issues will pave way for silicon nanomaterials to be modern day biosensor components. The wire- and electrode-less quartz–crystal-microbalance biosensors provide another platform for analyzing the interactions between biomolecules with high sensitivity. Pulsations of quartz oscillators were excited and detected by antennas through electromagnetic waves without any wire connections. This non-contacting precise measurement is a key feature for developing ultrahigh-sensitive detection of proteins in liquids using quartz–crystal biosensor-based instrumentation (Ogi, 2013). Considering the unique features of silica or quartz or glass materials, several new biosensors were developed with high-end technology for improving bioinstrumentation to biomedicine technology yet cost-effectiveness and biosafety requires attention (Ogi, 2013; Turner, 2013; Peng et al., 2014; Shen et al., 2014).

## Nanomaterials-Based Biosensors

Wide range of nanomaterials ranging from gold, silver, silicon, and copper nanoparticles, carbon-based materials, such as graphite, grapheme, and carbon nanotubes, are used for developing biosensor immobilization (Li et al., 2011; Zhou et al., 2012; Guo, 2013; Ko et al., 2013; Senveli and Tigli, 2013; Valentini et al., 2013; Lamprecht et al., 2014; Shen et al., 2014; Sang et al., 2015). In addition, nanoparticle-based materials provide great sensitivity and specificity for developing electrochemical and other types of biosensors. Among the metallic nanoparticles, gold nanoparticles have potential use because of their stability against oxidation (Hutter and Maysinger, 2013) and almost have no toxicity, while other nanoparticles like silver which oxidize and have toxic manifestation, if used for internally in medicine such as drug delivery. Largely, usage of nanomaterials for biosensors has potential challenges, which needs to be addressed if used for biomedicine (Su et al., 2011). Furthermore, nanoparticle-based signal amplification strategies have potential advantages and drawbacks (Ding et al., 2013). Nevertheless, nanomaterials are considered as critical components in bio-analytical devices simply for enhancing the sensitivity and detection limits for single molecule detection (Turner, 2013). In this context, it is worthwhile to mention the invention of platinum-based nanoparticles for electrochemical amplification with single label response for the detection of low concentration of DNA (Kwon and Bard, 2012). Similarly, semiconductor quantum dots and iron oxide nanocrystals having optical as well as magnetic properties can be effectively linked with tumor targeting ligands, such as monoclonal antibodies, peptides, or small molecules to target tumor antigens with high affinity and specificity (Nie et al., 2007). Quantum dots technology can be applied to understand the tumor microenvironment for therapeutics and also for the delivery of nano-medicine (Jain, 2013). Use of cantilever size (milli-, micro-, and nano-cantilevers) biosensors are even critically analyzed due to their application potential in various fields.

## Genetically Encoded or Synthetic Fluorescent Biosensors

Development of tagged biosensor using genetically encoded or synthetic fluorescence paved way to understand the biological process including various molecular pathways inside the cell (Kunzelmann et al., 2014; Oldach and Zhang, 2014; Randriamampita and Lellouch, 2014). In fact, the discovery of fluorescent-tagged antibodies was first developed to image fixed cells (Oldach and Zhang, 2014). This strategy indeed provided new ways to develop such sensors using biological proteins, small molecule binding to analytes and second messengers. More recently, fluorescent biosensors were developed for analyzing motor proteins using single molecule detection with specific analyte concentration (Kunzelmann et al., 2014). In spite of these advantages, the methodology of probe detection and analysis seems to be difficult. Invention of green fluorescent protein and other fluorescent proteins gave several advantages in terms of optical probe design and efficiency (Oldach and Zhang, 2014). Until last decade, genetically encoded biosensors targeting molecules related to energy production, reactive oxygen species and cAMP provided better understanding of mitochondrial physiology (De et al., 2014). Likewise, cGMP is an important signaling molecule and a drug target for cardiovascular system. In view of this, Förster resonance energy transfer (FRET)-based biosensors have been developed (Thunemann et al., 2014) for visualizing cGMP, cAMP, and Ca^2+^ in cells. Several of these sensors work efficiently in primary culture and live-cell *in vivo* imaging (Oldach and Zhang, 2014; Randriamampita and Lellouch, 2014). Quite a few key aspects have now been figured out for developing sensors for live-animal imaging. Optimizing such approaches, small-angle X-ray scattering for developing calcium sensors and fluorescence resonance energy transfer-probes for kinase sensing are being cited as best biosensors methods in modern physiology (Oldach and Zhang, 2014). By this way, several microbial and cell organelle-based biosensors were developed with specific targets (Su et al., 2012). As explained earlier, electrochemical, electromechanical, and optical biosensors are developed for detecting miRNA much more efficiently than other molecular techniques (Johnson and Mutharasan, 2014). Considering the advent of *in vivo* imaging with small molecule biosensors, a better understanding of cellular activity and many other molecules ranging from DNA, RNA, and miRNA have been identified (Khimji et al., 2013; Turner, 2013; Johnson and Mutharasan, 2014). Now the transformation in this field requires whole genome approach using better optical based genetic biosensors. It is also now widely accepted that optical-based biosensors with a combination of fluorescence and small molecules/nanomaterials have achieved greater success in terms of applications and sensitivity.

## Microbial Biosensors through Synthetic Biology and Genetic/Protein Engineering

More recent trend in environmental monitoring and bioremediation is to utilize state-of-the-art innovative technologies based on genetic/protein engineering and synthetic biology to program microorganisms with specific signal outputs, sensitivity, and selectivity. For example, live cell having enzyme activity to degrade xenobiotic compounds will have wider applications in bioremediation (Park et al., 2013). Similarly, microbial fuel-based biosensors have been developed with aim to monitor biochemical oxygen demand and toxicity in the environment. Bacteria have the potential of degrading the organic substrate and generating electricity for fermentation. Basically, the technology involves the usage of a bio-electrochemical device that controls the power of microbial respiration to convert organic substrates directly into electrical energy. In spite of these possibilities, the limitations are there in microbial biosensors due to low power density in terms of production and operating costs. Efforts are being made to significantly enhance the performance and cutting down the costs with new systemic approaches, wherein technologies have provided a platform to develop self-powered engineered microbial biosensors (Du et al., 2007; Sun et al., 2015). Another area of microbial biosensors reveal potential applications in pesticidal and heavy metal detection (Gutierrez et al., 2015) in which eukaryotic microorganisms have an edge over prokaryotic cells. This is primarily due to the advantage of developing whole cell biosensors (Gutierrez et al., 2015) with selective and sensitive applications related to the detection of heavy metal and pesticidal toxicity. Furthermore, the higher eukaryotic microbes can have wider sensitivity to different toxic molecules and have relevance to higher animals. Interestingly, the applications of microbial biosensors are diverse ranging from environmental monitoring to energy production. Innovative strategies will provide novel biosensors with high sensitivity than selectivity from microbial origin from eukaryotes to engineered prokaryotes. In future, these microbial biosensors (Du et al., 2007; Sun et al., 2015) will have wider applications in monitoring environmental metal pollution and sustainable energy production.

## Technological Comparison of Biosensors

In previous sections, we reviewed the different types of biosensors and their applications. In this part, we compared biosensors in terms of technology, specificity and detection limit, linear range, analysis time, cost and portability.

Innovations in electrochemical sensors with high-throughput methods focusing on detection limit, analysis time and portability provided large-scale consumer markets for inexpensive biosensors for glucose and pregnancy tests using anti-human chorionic gonadotropin immobilization strip with lateral-flow technology (Turner, 2013). Immobilization of analytes using polymers and nanomaterials are the key to improve the sensitivity and detection limit. In this viewpoint, lateral-flow technology allows direct delivery of samples to desired spot in order to create specific interactions instead of random. Much of the aforementioned biosensors have used this technology and, in fact, it has paved way for bio-fabrication using either contact or non-contact-based patterning. Use of nanomaterials like gold, silver, and silicon-based biofabrification yielded new methods. In addition, coating of polymers on these nanomaterials brought revolution in contact-based electrochemical sensing. One of the major advantages on these types of electrochemical sensors is sensitivity and specificity with real-time analysis. However, the limitations are the regenerative ability or long-term usage of polymers/other materials, yet reduction in cost makes such electrochemical sensors more affordable. Single analyte detection using contact-based sensing has tremendous advantages, for example, real-time measurement of molecules with high specificity. To enable this, FRET, bioluminescent resonance energy transfer, fluorescent-based, and surface plasmon resonance-based transducers have been introduced (Dias et al., 2014) to improve specificity and sensitivity in terms of single molecule detection. These techniques have limitations in multiple analyte detection due to signal emission overlap yet resonance energy transfer methods often demonstrated for multiple analyte detection, which is highly rewarded in clinical diagnosis due to difference in biomarkers between patients and related diseases. Use of micro- or nano-cantilevers as transducers in electrochemical sensor biofabrification is also having wider prospective in multiple analytes detection. In addition, non-contact-based sensors using 3D bioprinting using inkjet or laser direct writing provided better results. Nevertheless, the cost involved and customizable ability has high limitations in these methods. Interestingly, most of these high-throughput biosensors have been combined with electrochemical sensing for specific applications. Some of the most-notable sensitive, real-time and portable amperometric electrochemical biosensors have been developed (Kim et al., 2015) for disease diagnosis using body fluids. In general, electrochemical biosensors in combination with biofabrification have low detection limit for single analyte detection specificity with real-time analysis and also at an affordable cost considering the device portability.

Optic-based biosensors are next major technology in biosensing involving fiber-optic chemistry. Single molecule detection, for example, DNA or peptide, is best done using hydrogel-based cross-linking due to the advantage of having high loading capacity and hydrophilic nature. Later optical biosensor for DNA measurement have been developed (Kwon and Bard, 2012), which had wider applications in biomedicine and forensic science. Combination of biological materials, such as enzyme/substrate, antibody/antigen and nucleic acids, brought revolution to optical biosensor technology. In addition, it is also possible to incorporate microorganisms, animal or plant cells and tissue sections in the biosensing system. Recent advances in the molecular optoelectronics even offered optical biometric recognition systems. Integrated optics technology allows the incorporation of both passive and active optical components onto the same substrate for the development of minimized compact sensing devices using fabrication of multiple sensors on a single chip. In this context, high quality polymers provided hybrid systems for optical biosensors. In fact, optic-based biosensor technology got improved due to modern innovations in surface morphology analysis using high-end electron and atomic force microscopy. In spite of these, the detection limit of optical biosensors never had been close to femto-level considering the instrumentation cost and no device portability. To this end, modern optic technology involving nanomechanical biosensors based on microcantilevers and surface resonance technology have provided innovative DNA chips at least for real-time specific and sensitive analysis (Scheller et al., 2014; Wang et al., 2015; Zhang et al., 2015). Largely, advantages of optical biosensors include speedy swift analysis with the resistance of signal to electrical or magnetic interference and the potential for spectrum of information. But the main drawback is high cost due to certain instrumentation requirements. Other technical difficulties include intricacy in immobilization especially for bio-fabrication and requirement of sterilized environment need to be critically resolved to take full advantage of optical biosensors.

Bio-fabrication of mechanical devices provides better results for mass-based biosensors. Indeed, both electrochemical and optical biosensors make the most of this technology for inventing superior biosensors. Major advances in micro- and nanofabrication technologies (Arlett et al., 2011) enable the development of mechanical devices with nanosized moving parts. The ability to fabricate such structures using semiconductor processing procedures bridged biophysics and bioengineering principles toward the progress of practical micro- and nano-electromechanical biosensors that can be produced in large quantities (Arlett et al., 2011). Glass, silicon, and quartz materials have been used either after tagging with fluorescence or gold nanoparticles successfully. Though these biosensors have greater accuracy in terms of single molecule detection, low-cost mass production is less feasible (Peng et al., 2014; Shen et al., 2014). Many challenges still remain in mass-based sensors in terms of better capture agents to fabricate at nanoscale using microelectronic fabrication for high-throughput analysis. In this regard, it is worth mentioning the tremendous application potentials of semiconductor materials and quantum dot technology. Nonetheless, no existing technology in biosensors is able to execute simultaneous, real-time, quantitative assays for large arrays, but use of micro- and nanoscale cantilevers fabrication might make it possible (Dias et al., 2014).

Another major technical revolution in biosensors is the discovery of genetically encoded or synthetic fluorescent biosensors to analyze molecular mechanisms of biological processes (Kunzelmann et al., 2014; Oldach and Zhang, 2014; Randriamampita and Lellouch, 2014). Though these sensors have tremendous prospect for single molecule detection with specific analyte measurement, the methodology and probe preparation and detection are difficult and also require high-end instrumentation. In view of biomaterials, microbial fuel-based biosensors also have distinction due to high sensitivity and selectivity, yet mass production and genetic engineering methods to develop a microbial strain require complex procedures and high cost. However, one another advantage on microbial biosensors is the ability to use them as a tool for bioremediation, which have greater significance in terms of environmental monitoring. However, development and release of such genetically modified strain should be analyzed strictly with suitable governing laws and ethical clearance in addition to production cost management.

Taken together with the view of Scheller et al. (2014), development of different micro- and nano-biosensor platforms involving integrated technologies utilizing electrochemical or optic bio-electronic principles with a combination of biomolecules or biological materials, polymers, and nanomaterials are required in order to achieve highly sensitive and miniaturized devices.

## Current Research Trends, Future Challenges and Limitations of Biosensor Technology

Integrated strategies using multiple technologies ranging from electrochemical, electromechanical, and fluorescence-­cum-optical-based biosensors and genetically engineered microbes are modern methods for biosensor discoveries (Table 1). Some of these biosensors have tremendous application prospects in disease diagnosis and medicine (Table 2). As the demand and need for using biosensor for rapid analysis with cost-effectiveness require bio-fabrication that will pave way to identify cellular to whole animal activity with a detection limit of high accuracy for single molecules. Next, the biosensors should be targeted to work under multiplex conditions. In that situation, both 2D and 3D detection are required with sophisticated transducers for targeting and quantifying small analytes of interest (Dias et al., 2014). In this, several milestone discoveries were made with contact or non-contact-based patterning at different levels. Next level of development should aim for discovering more robust regenerative biosensors for long-term use. If this happens, new diagnostic biosensors can be developed for therapeutics, which will help both clinicians and patients in a long run for more integrative understanding of diseases and therapy. In view of this, fluorescence resonance energy transfer-based biosensor provided excellent diagnostic procedure for assessing the efficacy of imatinib treatment in chronic myeloid leukemia (Fracchiolla et al., 2013). Current use of aptamers, affibodies, peptide arrays, and molecularly imprinted polymers are classical examples for prospective research approaches in this field (Citartan et al., 2013; Abe et al., 2014; Verma and Bhardwaj, 2015). Little success is also achieved with few potential molecules for novel therapeutic, antimicrobial, and drug delivery. Invention on this line leads to discovery of electrochemical biosensors as reliable analytical devices for pathogen detection of avian influenza virus in the complex matrices (Grabowska et al., 2014). More recent report revealed potential applications of affinity-based biosensors in sport medicine and doping control analysis (Mazzei et al., 2014). More recently, variety of wearable electrochemical biosensors were reviewed in detail for real-time non-invasive screening of electrolytes and metabolites in body fluid as indicators of a wearer’s health status (Bandodkar and Wang, 2014). Another interesting application is assessing the meat and fish quality by using hypoxanthine biosensors by fabrication (Lawal and Adeloju, 2012). Development in biosensors for the detection of biological warfare agents ranging from bacteria, virus, and toxins is often attempted using various devices of biosensors ranging from electrochemical, nucleic acid, optical and piezoelectric, which will have immense applications in military and health as well as defense and security (Kumar and Rani, 2013). Taken together, combination of nanomaterials and polymers with various types of biosensors will provide hybrid devices for better usage in the aforementioned applications (Citartan et al., 2013; Turner, 2013). In addition, scientific advancement in developing microbial biosensors with synthetic biology approach will largely contribute for environmental monitoring and energy demand (Sun et al., 2015). The authors of this report further signified the importance of using microbial fuel cells for developing a method of water treatment and as power sources for environmental sensors. On a broader perspective, we highlighted the type of biosensors, potential application and characteristics like analyte detection ability, analysis time, portability, cost and customization (Table 3).

**Table 1:** **List of biosensors with principle, applications, and bibliography**.

Sl. No.	Type	Principle	Applications	Bibliography (review/original article)
1.	Glucose oxidase electrode based biosensor	Electrochemistry using glucose oxidation	Analysis of glucose in biological sample	Clark and Lyons (1962)
2.	HbA1c biosensor	Electrochemistry using ferroceneboronic acid	Robust analytical method for measuring glycated hemoglobin	Wang et al. (2014)
3.	Uric acid biosensor	Electrochemistry	For detection of clinical abnormalities or diseases	Erden and Kilic (2013) and Kim et al. (2015)
4.	Acetylcholinesterase inhibition-based biosensors	Electrochemistry	Understanding pesticidal impact	Pundir and Chauhan (2012)
5.	Piezoelectric biosensors	Electrochemistry	Detecting organophosphate and carbamate	Marrazza (2014)
6.	Microfabricated biosensor	Optical/visual biosensor using cytochrome P450 enzyme	For drug development	Schneider and Clark (2013)
7.	Hydrogel (polyacrylamide)-based biosensor	Optical/visual biosensor	Biomolecular immobilization	Khimji et al. (2013)
8.	Silicon biosensor	Optical/visual/fluorescence	Bioimaging, biosensing and cancer therapy	Peng et al. (2014) and Shen et al. (2014)
9.	Quartz–crystal biosensor	Electromagnetic	For developing ultrahigh-sensitive detection of proteins in liquids	Ogi (2013)
10.	Nanomaterials-based biosensors	Electrochemical or optical/visual/fluorescence	For multifaceted applications including biomedicine, for example diagnostic tools	Li et al. (2011), Kwon and Bard (2012), Zhou et al. (2012), Guo (2013), Hutter and Maysinger (2013), Ko et al. (2013), Senveli and Tigli (2013), Valentini et al. (2013), Lamprecht et al. (2014) and Sang et al. (2015)
11.	Genetically encoded or fluorescence-tagged biosensor	Fluorescence	For understanding biological process including various molecular systems inside the cell	Randriamampita and Lellouch (2014), Oldach and Zhang (2014), Kunzelmann et al. (2014) and Wang et al. (2015)
12.	Microbial fuel cell-based biosensors	Optical	To monitor biochemical oxygen demand and toxicity in the environment and heavy metal and pesticidal toxicity	Gutierrez et al. (2015) and Sun et al. (2015)

**Table 2:** **Use of biosensors in disease diagnosis**.

Sl. No.	Biosensor(s)	Disease diagnosis or medical applications
1.	Glucose oxidase electrode based biosensor and HbA1c biosensor	Diabetes
2.	Uric acid biosensor	Cardiovascular and general disease diagnosis
3.	Microfabricated biosensor	Optical corrections
4.	Hydrogel (polyacrylamide)-based biosensor	Regenerative medicine
5.	Silicon biosensor	Cancer biomarker development and applications
6.	Nanomaterials-based biosensors	For therapeutic applications

**Table 3:** **Type of biosensors with applications and characteristics**.

Sl. No.	Type of biosensor	Applications	Characteristics			
			Analyte detection: single (S) or multiple (M)	Real-time (***) & sensitivity (***)	Portability (yes/no)	Cost ($$$$) & customization (***)
1.	Electrochemical (traditional/old)	Disease diagnosis	S	No & *	No	$ & *
2.	Electrochemical with biofabrification (modern)	Disease diagnosis and environmental monitoring	S & M	*** & **	Yes	$$ & ***
3.	Optical/visual/fluorescence	Drug development, bioimaging and biosensing	S	*** & ***	No	$$$ & ***
4.	Optical/visual/fluorescence with biofabrification	Drug development, bioimaging and biosensing	M	*** & ***	No	$$$$ & ***
5.	Microbial	Energy production and environmental sensing	S	* & **	Yes	$$ & **
6.	Electromagnetic	Protein biology	S	** & **	No	$ & *

In conclusion, the development of biosensors mostly relies on sensitivity, specificity, non-toxicity, small molecule detection and cost-effectiveness. Considering these characteristics will eventually address critical parameters required and the concern related to major limitations of the biosensor technology. Some of the advancement are seen in electrochemical sensors in combination with nanomaterials results in new types of biosensors (Kwon and Bard, 2012; Bandodkar and Wang, 2014). In this view point, it is worth mentioning the invention of “electronic skin” in the form of printed temporary transfer tattoo electrochemical biosensors for physiological and security detection of chemical ingredients (Windmiller et al., 2012a,b). Overall, better combination of biosensing and bio-fabrication with synthetic biology approaches using either electrochemical or optic or bio-electronic principles or a combination of all these will be the key for successful development of powerful biosensors for modern era.

## Author Contributions

First author conceived and contributed largely for the review while all other authors contributed in organizing.

## Conflict of Interest Statement

The authors declare that the research was conducted in the absence of any commercial or financial relationships that could be construed as a potential conflict of interest.

## References

[B1] Abe K. Yoshida W. Ikebukuro K. (2014). Electrochemical biosensors using aptamers for theranostics. Adv. Biochem. Eng. Biotechnol. 140, 183–202. 10.1007/10_2013_226 23873093

[B2] Arlett J. L. Myers E. B. Roukes M. L. (2011). Comparative advantages of mechanical biosensors. Nat. Nanotechnol. 6, 203–215. 10.1038/nnano.2011.44 21441911 PMC3839312

[B3] Bahadir E. B. Sezginturk M. K. (2015). Electrochemical biosensors for hormone analyses. Biosens. Bioelectron. 68, 62–71. 10.1016/j.bios.2014.12.054 25558874

[B4] Bandodkar A. J. Wang J. (2014). Non-invasive wearable electrochemical sensors: a review. Trends Biotechnol. 32, 363–371. 10.1016/j.tibtech.2014.04.005 24853270

[B5] Citartan M. Gopinath S. C. Tominaga J. Tang T. H. (2013). Label-free methods of reporting biomolecular interactions by optical biosensors. Analyst 138, 3576–3592. 10.1039/c3an36828a 23646346

[B6] Clark L. C. Jr. Lyons C. (1962). Electrode systems for continuous monitoring in cardiovascular surgery. Ann. N. Y. Acad. Sci. 102, 29–45. 10.1111/j.1749-6632.1962.tb13623.x 14021529

[B7] De M. R. Carimi F. Frommer W. B. (2014). Mitochondrial biosensors. Int. J. Biochem. Cell Biol. 48, 39–44. 10.1016/j.biocel.2013.12.014 24397954

[B8] Dias A. D. Kingsley D. M. Corr D. T. (2014). Recent advances in bioprinting and applications for biosensing. Biosensors (Basel) 4, 111–136. 10.3390/bios4020111 25587413 PMC4264374

[B9] Ding L. Bond A. M. Zhai J. Zhang J. (2013). Utilization of nanoparticle labels for signal amplification in ultrasensitive electrochemical affinity biosensors: a review. Anal. Chim. Acta 797, 1–12. 10.1016/j.aca.2013.07.035 24050664

[B10] Du Z. Li H. Gu T. (2007). A state of the art review on microbial fuel cells: a promising technology for wastewater treatment and bioenergy. Biotechnol. Adv. 25, 464–482. 10.1016/j.biotechadv.2007.05.004 17582720

[B11] Erden P. E. Kilic E. (2013). A review of enzymatic uric acid biosensors based on amperometric detection. Talanta 107, 312–323. 10.1016/j.talanta.2013.01.043 23598228

[B12] Fracchiolla N. S. Artuso S. Cortelezzi A. (2013). Biosensors in clinical practice: focus on oncohematology. Sensors (Basel) 13, 6423–6447. 10.3390/s130506423 23673681 PMC3690064

[B13] Grabowska I. Malecka K. Jarocka U. Radecki J. Radecka H. (2014). Electrochemical biosensors for detection of avian influenza virus – current status and future trends. Acta Biochim. Pol. 61, 471–478. 25180225

[B14] Gruhl F. J. Rapp B. E. Lange K. (2013). Biosensors for diagnostic applications. Adv. Biochem. Eng. Biotechnol. 133, 115–148. 10.1007/10_2011_130 22223139

[B15] Guo X. (2013). Single-molecule electrical biosensors based on ­single-walled carbon nanotubes. Adv. Mater. 25, 3397–3408. 10.1002/adma.201301219 23696446

[B16] Gutierrez J. C. Amaro F. Martin-Gonzalez A. (2015). Heavy metal whole-cell biosensors using eukaryotic microorganisms: an updated critical review. Front. Microbiol. 6:48. 10.3389/fmicb.2015.00048 25750637 PMC4335268

[B17] Hamidi-Asl E. Palchetti I. Hasheminejad E. Mascini M. (2013). A review on the electrochemical biosensors for determination of microRNAs. Talanta 115, 74–83. 10.1016/j.talanta.2013.03.061 24054564

[B18] Harris J. M. Reyes C. Lopez G. P. (2013). Common causes of glucose oxidase instability in in vivo biosensing: a brief review. J. Diabetes Sci. Technol. 7, 1030–1038. 23911187 10.1177/193229681300700428 PMC3879770

[B19] Hutter E. Maysinger D. (2013). Gold-nanoparticle-based biosensors for detection of enzyme activity. Trends Pharmacol. Sci. 34, 497–507. 10.1016/j.tips.2013.07.002 23911158

[B20] Jain R. K. (2013). Normalizing tumor microenvironment to treat cancer: bench to bedside to biomarkers. J. Clin. Oncol. 31, 2205–2218. 10.1200/JCO.2012.46.3653 23669226 PMC3731977

[B21] Johnson B. N. Mutharasan R. (2014). Biosensor-based microRNA detection: techniques, design, performance, and challenges. Analyst 139, 1576–1588. 10.1039/c3an01677c 24501736

[B22] Khimji I. Kelly E. Y. Helwa Y. Hoang M. Liu J. (2013). Visual optical biosensors based on DNA-functionalized polyacrylamide hydrogels. Methods 64, 292–298. 10.1016/j.ymeth.2013.08.021 23978515

[B23] Kim J. Imani S. de Araujo W. R. Warchall J. Valdes-Ramirez G. Paixao T. R. (2015). Wearable salivary uric acid mouthguard biosensor with integrated wireless electronics. Biosens. Bioelectron. 74, 1061–1068. 10.1016/j.bios.2015.07.039 26276541 PMC4718709

[B24] Ko P. J. Ishikawa R. Sohn H. Sandhu A. (2013). Porous silicon platform for optical detection of functionalized magnetic particles biosensing. J. Nanosci. Nanotechnol. 13, 2451–2460. 10.1166/jnn.2013.7406 23763119

[B25] Kumar H. Rani R. (2013). Development of biosensors for the detection of biological warfare agents: its issues and challenges. Sci. Prog. 96, 294–308. 10.3184/003685013X13777066241280 24244972 PMC10365506

[B26] Kunzelmann S. Solscheid C. Webb M. R. (2014). Fluorescent biosensors: design and application to motor proteins. EXS 105, 25–47. 10.1007/978-3-0348-0856-9_2 25095989

[B27] Kwon S. J. Bard A. J. (2012). DNA analysis by application of Pt nanoparticle electrochemical amplification with single label response. J. Am. Chem. Soc. 134, 10777–10779. 10.1021/ja304074f 22702801

[B28] Lamprecht C. Hinterdorfer P. Ebner A. (2014). Applications of biosensing atomic force microscopy in monitoring drug and nanoparticle delivery. Expert. Opin. Drug Deliv. 11, 1237–1253. 10.1517/17425247.2014.917078 24809228

[B29] Lawal A. T. Adeloju S. B. (2012). Progress and recent advances in fabrication and utilization of hypoxanthine biosensors for meat and fish quality assessment: a review. Talanta 100, 217–228. 10.1016/j.talanta.2012.07.085 23141330

[B30] Li M. Li R. Li C. M. Wu N. (2011). Electrochemical and optical biosensors based on nanomaterials and nanostructures: a review. Front. Biosci. (Schol Ed) 3:1308–1331. 10.2741/228 21622273

[B31] Long F. Zhu A. Shi H. (2013). Recent advances in optical biosensors for environmental monitoring and early warning. Sensors (Basel) 13, 13928–13948. 10.3390/s131013928 24132229 PMC3859100

[B32] Marrazza G. (2014). Piezoelectric biosensors for organophosphate and carbamate pesticides: a review. Biosensors (Basel) 4, 301–317. 10.3390/bios4030301 25587424 PMC4264360

[B33] Mazzei F. Antiochia R. Botre F. Favero G. Tortolini C. (2014). Affinity-based biosensors in sport medicine and doping control analysis. Bioanalysis 6, 225–245. 10.4155/bio.13.308 24423598

[B34] Mello L. D. Kisner A. Goulart M. O. Kubota L. T. (2013). Biosensors for antioxidant evaluation in biological systems. Comb. Chem. High Throughput Screen. 16, 109–120. 10.2174/138620713804806265 23092169

[B35] Nie S. Xing Y. Kim G. J. Simons J. W. (2007). Nanotechnology applications in cancer. Annu. Rev. Biomed. Eng. 9, 257–288. 10.1146/annurev.bioeng.9.060906.152025 17439359

[B36] Ogi H. (2013). Wireless-electrodeless quartz-crystal-microbalance biosensors for studying interactions among biomolecules: a review. Proc. Jpn. Acad. Ser. B Phys. Biol. Sci. 89, 401–417. 10.2183/pjab.89.401 24213205 PMC3865356

[B37] Oldach L. Zhang J. (2014). Genetically encoded fluorescent biosensors for live-cell visualization of protein phosphorylation. Chem. Biol. 21, 186–197. 10.1016/j.chembiol.2013.12.012 24485761 PMC4050661

[B38] Park K. Jung J. Son J. Kim S. H. Chung B. H. (2013). Anchoring foreign substances on live cell surfaces using Sortase A specific binding peptide. Chem. Commun. (Camb) 49, 9585–9587. 10.1039/c3cc44753g 24018381

[B39] Peng F. Su Y. Zhong Y. Fan C. Lee S. T. He Y. (2014). Silicon nanomaterials platform for bioimaging, biosensing, and cancer therapy. Acc. Chem. Res. 47, 612–623. 10.1021/ar400221g 24397270

[B40] Pundir C. S. Chauhan N. (2012). Acetylcholinesterase inhibition-based biosensors for pesticide determination: a review. Anal. Biochem. 429, 19–31. 10.1016/j.ab.2012.06.025 22759777

[B41] Randriamampita C. Lellouch A. C. (2014). Imaging early signaling events in T lymphocytes with fluorescent biosensors. Biotechnol. J. 9, 203–212. 10.1002/biot.201300195 24166755

[B42] Sang S. Wang Y. Feng Q. Wei Y. Ji J. Zhang W. (2015). Progress of new label-free techniques for biosensors: a review. Crit. Rev. Biotechnol. 15, 1–17. 10.3109/07388551.2014.991270 25608959

[B43] Scheller F. W. Yarman A. Bachmann T. Hirsch T. Kubick S. Renneberg R. (2014). Future of biosensors: a personal view. Adv. Biochem. Eng. Biotechnol. 140, 1–28. 10.1007/10_2013_251 24196315

[B44] Schneider E. Clark D. S. (2013). Cytochrome P450 (CYP) enzymes and the development of CYP biosensors. Biosens. Bioelectron. 39, 1–13. 10.1016/j.bios.2012.05.043 22809523

[B45] Senthilkumaran B. (2015). Pesticide- and sex steroid analogue-induced endocrine disruption differentially targets hypothalamo-hypophyseal-gonadal system during gametogenesis in teleosts – a review. Gen. Comp. Endocrinol. 219, 136–142. 10.1016/j.ygcen.2015.01.010 25637674

[B46] Senveli S. U. Tigli O. (2013). Biosensors in the small scale: methods and technology trends. IET Nanobiotechnol. 7, 7–21. 10.1049/iet-nbt.2012.0005 23705288

[B47] Shen M. Y. Li B. R. Li Y. K. (2014). Silicon nanowire field-effect-transistor based biosensors: from sensitive to ultra-sensitive. Biosens. Bioelectron. 60, 101–111. 10.1016/j.bios.2014.03.057 24787124

[B48] Su L. Jia W. Hou C. Lei Y. (2011). Microbial biosensors: a review. Biosens. Bioelectron. 26, 1788–1799. 10.1016/j.bios.2010.09.005 20951023

[B49] Su T. Zhang Z. Luo Q. (2012). Ratiometric fluorescence imaging of dual bio-molecular events in single living cells using a new FRET pair mVenus/mKOkappa-based biosensor and a single fluorescent protein biosensor. Biosens. Bioelectron. 31, 292–298. 10.1016/j.bios.2011.10.034 22088261

[B50] Sun J. Z. Peter K. G. Si R. W. Zhai D. D. Liao Z. H. Sun D. Z. (2015). Microbial fuel cell-based biosensors for environmental monitoring: a review. Water Sci. Technol. 71, 801–809. 10.2166/wst.2015.035 25812087

[B51] Thunemann M. Schmidt K. de W. C. Han X. Jain R. K. Fukumura D. (2014). Correlative intravital imaging of cGMP signals and vasodilation in mice. Front. Physiol. 5:394. 10.3389/fphys.2014.00394 25352809 PMC4196583

[B52] Turner A. P. (2013). Biosensors: sense and sensibility. Chem. Soc. Rev. 42, 3184–3196. 10.1039/c3cs35528d 23420144

[B53] Valentini F. Galache F. L. Tamburri E. Palleschi G. (2013). Single walled carbon nanotubes/polypyrrole-GOx composite films to modify gold microelectrodes for glucose biosensors: study of the extended linearity. Biosens. Bioelectron. 43, 75–78. 10.1016/j.bios.2012.11.019 23277343

[B54] Verma N. Bhardwaj A. (2015). Biosensor technology for pesticides – a review. Appl. Biochem. Biotechnol. 175, 3093–3119. 10.1007/s12010-015-1489-2 25595494

[B55] Wang B. Takahashi S. Du X. Anzai J. (2014). Electrochemical biosensors based on ferroceneboronic acid and its derivatives: a review. Biosensors (Basel) 4, 243–256. 10.3390/bios4030243 25587421 PMC4264357

[B56] Wang J. Chen G. Jiang H. Li Z. Wang X. (2013). Advances in nano-scaled biosensors for biomedical applications. Analyst 138, 4427–4435. 10.1039/c3an00438d 23748648

[B57] Wang S. Poon G. M. Wilson W. D. (2015). Quantitative investigation of protein-nucleic acid interactions by biosensor surface plasmon resonance. Methods Mol. Biol. 1334, 313–332. 10.1007/978-1-4939-2877-4_20 26404159 PMC4640367

[B58] Windmiller J. R. Bandodkar A. J. Parkhomovsky S. Wang J. (2012a). Stamp transfer electrodes for electrochemical sensing on non-planar and oversized surfaces. Analyst 137, 1570–1575. 10.1039/c2an35041f 22363933

[B59] Windmiller J. R. Bandodkar A. J. Valdes-Ramirez G. Parkhomovsky S. Martinez A. G. Wang J. (2012b). Electrochemical sensing based on printable temporary transfer tattoos. Chem. Commun. (Camb) 48, 6794–6796. 10.1039/c2cc32839a 22669136 PMC3387561

[B60] Zhang Z. Liu J. Qi Z. M. Lu D. F. (2015). In situ study of self-assembled nanocomposite films by spectral SPR sensor. Mater. Sci. Eng. C Mater. Biol. Appl. 51, 242–247. 10.1016/j.msec.2015.02.026 25842131

[B61] Zhou Y. Chiu C. W. Liang H. (2012). Interfacial structures and properties of organic materials for biosensors: an overview. Sensors (Basel) 12, 15036–15062. 10.3390/s121115036 23202199 PMC3522952

